# Micro-CT Imaging of Denatured Chitin by Silver to Explore Honey Bee and Insect Pathologies

**DOI:** 10.1371/journal.pone.0027448

**Published:** 2011-11-16

**Authors:** Peter R. Butzloff

**Affiliations:** Honey Bee Research Institute and Nature Center, Saint David, Maine, United States of America; Ghent University, Belgium

## Abstract

**Background:**

Chitin and cuticle coatings are important to the environmental and immune defense of honey bees and insect pollinators. Pesticides or environmental effects may target the biochemistry of insect chitin and cuticle coating. Denaturing of chitin involves a combination of deacetylation, intercalation, oxidation, Schweiger-peeling, and the formation of amine hydrochloride salt. The term “denatured chitin” calls attention to structural and property changes to the internal membranes and external carapace of organisms so that some properties affecting biological activities are diminished.

**Methodology/Principal Findings:**

A case study was performed on honey bees using silver staining and microscopic computer-tomographic x-ray radiography (micro-CT). Silver nitrate formed counter-ion complexes with labile ammonium cations and reacted with amine hydrochloride. Silver was concentrated in the peritrophic membrane, on the abdomen, in the glossa, at intersegmental joints (tarsi), at wing attachments, and in tracheal air sacs. Imaged mono-esters and fatty acids from cuticle coating on external surfaces were apparently reduced by an alcohol pretreatment.

**Conclusions/Significance:**

The technique provides 3-dimensional and sectional images of individual honey bees consistent with the chemistries of silver reaction and complex formation with denatured chitin. Environmental exposures and influences such as gaseous nitric oxide intercalant, trace oxidants such as ozone gas, oligosachharide salt conversion, exposure to acid rain, and chemical or biochemical denaturing by pesticides may be studied using this technique. Peritrophic membranes, which protect against food abrasion, microorganisms, and permit efficient digestion, were imaged. Apparent surface damage to the corneal lenses of compound eyes by dilute acid exposure consistent with chitin amine hydrochloride formation was imaged. The technique can contribute to existing insect pathology research, and may provide an additional tool for research on CCD.

## Introduction

Insect chitin and cuticle coating form major protective barriers against abrasion, chemical attack, and microbial disease. Chitin chemistry research may offer considerable advances toward assisting in the continued survival and well-being of the European honey bee, *Apis mellifera*. The antimicrobial and anti-fungal activity of chitin, chitosan, its derivatives, and oligomers arises from amine positive charges [1]. Environmental effects leading to the breach of cuticle coatings and the denaturing of chitin are associated with a reduction of chemical resistance, antimicrobial mechanisms at neutral pH [2], and digestive or immune response reduction in insects. Imaging and identifying the location of such changes may lead researchers to better understand if and how insect pathologies and environmental effects may be links to insect pollinator decline and honey bee colony collapse disorder (CCD).

Some environmentally caused pollinator declines [3] have been attributed to habitat loss, habitat fragmentation, loss of genetic diversity, foreign parasite introduction, climate change [4], [5], and even dietary deficiencies [6]. Pesticides often target the biochemistry of insect chitin [7], [8]. Additional susceptibilities include viral and fungal spore combinations such as iridovirus with microsporidian [9] as well as other infectious microbes [10]. A prevailing mechanism and root cause leading to so many complex and interrelated variables has not yet been found. Many causes have been suggested and later ruled out as insufficiently comprehensive.

The health and well-being of all pollinating insects directly affects the global food supply production; any decline in any of the pollinating insects should be of concern to agriculture. 100 crop species provide 90% of worldwide food. About 75% of these crops rely upon natural pollinators [11], and 71 of these crops rely on pollination by various species of bees [12]. An estimated 33% of these 100 crops rely upon the pollination services of the European honey bee. A comparison between the reported natural pollinators food value estimate, and the reported honey bee specific food value estimate, suggests pollinators other than honey bees constitute 42% of the current worldwide contribution to food production. Honey bees have experienced severe and sharp global declines since 2006 [13]. These trends continue to remain poorly understood and therefore poorly managed. The recent magnitude and rapid honey bee loss differs from past circumstances because it has become clear that bees are failing to return to many hives. The disorder results when large numbers of hives are lost in a short time. The choice of the word “disorder” rather than “disease” in hive or colony loss reflects the earliest understanding that some factor other than a specific disease or parasite must be involved in the sudden global decline of honey bees. Massive sudden losses of managed honey bee colonies may be associated with wild pollinator declines [14].

Speculation that environmental influences may compromise insect immune function leads logically toward a rigorous consideration of chemistry that may impair chitin immune expression or impair its resistance by breaching the protective cuticle coating either from the inside or from the outside of the insect. Insect mortality must be considered from bottom-up (host quality) and top-down (natural enemies) effects [15]. Environmental stress, plant nutritional composition, and locally expressed biosynthesis can alter the plant-insect herbivore interaction. Enhanced distributions of relatively shorter chained waxy alcohols (C18–C20) were found at the head of honey bees as compared with lengths of (C22–C32) found at the abdomen [16]. In some plants, enhanced relative distributions of shorter length n-alkane waxy cuticle coatings (C18–C23) result after exposure to gaseous and particulate pollutants [17]. These and other environmental influences may combine to measurably alter insect chitin chemistry.

Chitin was first chemically isolated and recovered in 1859 [18]. Chitin is a homopolymer composed mostly of 2-acetamide-2-deoxy-d-glucopyranose, and has a statistical mean molecular mass exceeding 1 mega-Daltons (mDa). This corresponds to a molecular chain length of approximately 5000 monomeric molecular units. Chitin has three known crystal forms [19]. The molecular structure of chitin in insect pollinators is in the beta crystal form.

### Deacetylation of Chitin

The N-deacetylated derivative of chitin is named chitosan. Chitosan is an industrially significant complex sugar biopolymer derivative known as a polysaccharide. Chitosan is used in a wide range of medical applications such as bandages and artificial bone scaffolding materials for reconstructive surgery. Chitosan is a heteropolymer of β (1–4) 2-acetamido-2-deoxy-β-D-glucopyranose (N-acetylglucosamine) and 2-amino-2-deoxy-β-D-glucopyranose (D-glucosamine) units. These monomer units are usually distributed randomly throughout the biopolymer.

Chitin loses N-acetyl groups along its polymer chain when exposed to acidic or caustic pH conditions. The loss of N-acetyl groups forms deacetylated D-glucosamine units in chitin. This exposes primary amine groups of polar character that attract water and swell chitin with intercalated water molecules. The amino groups take on a positive surface charge in the presence of acid. A greater degree of deacetylation causes a correspondingly greater cationic charge density. Greater than 50% conversion of N-acetyl-d-glucosamine units to d-glucosamine classifies the polysaccharide as chitosan; less than this proportion defines chitin [20]. Natural chitin usually has a small degree of deacetylation. Chitosan is easily wet by water, and is hydrophilic. Chitin is less able to be wet by water, and is hydrophobic. N-acetyl groups are removed by acids less effectively than alkaline treatments [21], [22]. Enzymatic deacetylation of chitin is achieved by certain fungi *in-vivo* [23]. Gel electrophoresis is the preferred assay for deacetylation of chitin and chitosan [24], [25] however this analysis is destructive, and does not assay the insoluble chitin fraction. Infra-red analysis of deacetylation is a non-destructive qualitative assay capable of supplementing field work linked to chitin dysfunction; however, it has not yet been reported for use in creating a map of external chitin chemical properties in insects.

The hydrolysis of chitin, its dissolution and deacetylation, and the potential for regeneration of chitin from chitosan are mediated by amidization, vitrification, and network formation. These transformations are best understood by time–temperature–transformation (TTT) diagrams [26].

### Depolymerization, Salt Conversion, and Solvation of Chitin

Depolymerization of chitin and chitosan occurs by cleaving the molecule at the O-glycosidic linkage without changing the degree of deacetylation. Charge density is not altered by depolymerization. Deacetylation of insoluble chitin into slightly soluble chitosan, and molecular weight reduction by depolymerization increases permeability and swelling by water, and reduces chitin crystallinity, resulting in a reduction of mechanical properties [27].

Transition element ions assist oxidative–reductive depolymerization of chitin by enzymes [28]. Non-specific proteases are capable of reducing chitin molecular weight to about 4.1 - 10.0 kDa [29]. White linearly polarized light increases enzymatic depolymerization of chitin by as much as 77% [30].

Chitinous biopolymers are industrially solvated and depolymerized in acids such as hydrochloric acid [31], sulfuric acid [32], nitrous acid [33], [34], phosphoric acid [35], and acetic acid [36]. These serve as singular introductions to the mechanisms of acid hydrolysis of chitin to glucosamine hydrochloride or its sulfate, nitrate, phosphate, acetate, and similar preparations by salt conversion. Acidic rain (or acid rain) is often a complex mixture of nitrous, nitric, sulfurous and sulfuric acids which may combine to lower the pH of rainwater to less than 5. When low pH exposure to chitin is followed by or combined with exposure to common salt-containing dust or salt fogs, this can result in amine salt conversion of chitosan to chitooligosaccharide hydrochloride. These salts interfere with ion conduction and antimicrobial efficacy arising from the neutralization of amine charge expression [20].

Extensive industrial solvation of chitin is only achieved at high temperatures, 50% deacetylation or greater, and at pH<6 [37]. Ultrasonic energy, together with acids, accelerates the depolymerization of chitin by creation of free radicals [38], [39]. Ozonolysis in water is more effective at chitin depolymerization than ultraviolet radiation [40].

### Reactive Oxygen Species (ROS) effects on Chitin

Chitin relies on the protonated amine positive charge of deacetylated regions to maintain an insect's protective cuticle coating. This function is achieved by the attraction of amino groups on chitin external surfaces to negative charges in fatty acids and mono-esters. This property has found use in neutraceuticals to bind with cholesterol and lipids to sequester dietary fat [41]. Protective mono-ester production on chitin peaks in worker bees at 15 days, whereas larvae and aged insects tend to produce long chain hydrocarbons instead [42]. Esters and fatty acids serve as a barrier to harmful reactive oxygen species (ROS) such as ozone that react with amine groups in chitin. ROS reaction with chitin forms Schiff bases that can be imaged by an increase in fluorescence in the range of 430–470 nm. Evidence of ozone reaction with chitin was found, especially at the honey bee thoracic region [43], forming small insoluble particles. These particles had fluorescence that was stable on heating without sublimation in a vacuum to 120°C. Because these chitin oxidation products were insoluble, they defied identification by mass spectroscopy or liquid chromatography. The method of soluble fluorescent dyes can identify soluble protein oxidation products and can be used to distinguish between external hydrophobic and hydrophilic regions on chitin surfaces [44].

The most frequently used biomarker of oxidative damage are carbonyl chromophores formed on protein residues after they are chemically damaged by oxidative reaction, but these were not found in honey bees exposed to significant oxidative stress [45]. These reports leave open the question of how ozone or oxidative stress is associated with insoluble fluorescent particles. It may be insufficient to look for soluble protein damage in a naturally insoluble chitin. New methods are needed to assay chitin for damage without destroying insect structures by dissolving them. Dissolving chitin sacrifices important information about the location of chemical and material property changes.

Ozone gas is significantly more effective at cleaving and depolymerizing chitosan than ozonolysis by free-radical reaction in water, providing a key link in understanding rapid and significant changes to chitin properties [46]. Ozone is a common ground level environmental pollutant and a strong oxidant. Chemical oxidation of plants by ozone increases plant susceptibility to disease, epidemics, and pests [47]. Ozone pollutant concentration in the lower atmosphere (troposphere) is projected to double by the year 2100, and therefore exceed all current international environmental limits [48], [49]. One major source of ground level ozone is from the decomposition of nitric oxide pollutants, which is increasing from 0.5 to 2% per year. About 0.15 to 0.51 ppm is currently the ozone peak concentration range in American cities. Los Angles, California, declares its Smog Alert Level 1 at 0.500 ppm, Alert 2 at 1.00 ppm, and Alert 3 at 1.500 ppm [50].

High regional variations of concentrated ozone in low-level atmospheric pockets of stagnant air have been reported in ground-based studies [51], especially during morning and evening twilight. Ozone gas is more than twice as dense as ordinary atmosphere. As atmospheric winds cease, intermingled ozone descends toward ground level where foraging bees become active at dawn.

Reactive oxygen species (ROS) are known to shorten lifespan in all organisms via genetic telomere length reduction. Genetic evidence of telomere reduction in honey bees was proposed to be a causative mechanism in CCD [52]. One or more ROS may be capable of causing chemical damage to bees, but must still be properly identified, as not all ROS may play a significant role in causing telomere damage. Nitric oxides and ozone exposure are two man-made gas phase pollutants associated with ROS in living organisms. Chitosan forms an amine adduct with anionic [NONO]- groups as ROS counter-ions [53], [54]. Surprisingly, no toxic limiting dosage (LD) studies of ozone or nitric oxides have been directed at bees or insect pollinators to date.

Long-term dissolved ozone toxicity exposure testing of farmed Pacific white shrimp in sea water occurs at levels between 0.10 to 0.15 ppm [55]. One significant observation reported is the visible effect of ozone to produce large gaps between exterior chitin and internal shrimp flesh. Formation of large abscesses indicates the presence of “soft shell syndrome” in shrimp. This observation has not yet been reported in honey bees or insect pollinators, even though gas phase ozone is more reactive to chitin than dissolved ozone-water. In a comparison of chitin characteristics, honey bee chitin at 96% acetylation has greater protein content than shrimp chitin at 95% acetylation [56]. Soluble extracts of protein content decreases with age in honey bees [57]. Protein is present in bee chitin, but the effect of protein loss due to ozone exposure has not been established, and “soft shell syndrome,” has not yet been associated with any species of insect.

### Denaturing of Chitin

Crystalline order is established in chitin gradually over time. Short term changes in the volume of chitin due to acetylation or deacetylation may act to disrupt its crystalline structure either by swelling it or by shrinking it. Protonating chitin amino groups and increasing the degree of acetylation disrupts crystalline order while broadening polysaccharide polymer solubility in pH neutral environments [58]. Chitin crystallinity decreases as amine groups become exposed through conversion into amorphous chitin with increased degrees of deacetylation. These swelling effects were clarified to be a charge-transfer mediated intercalation process by using an iodine agent as a chemical probe [59]. Together, steric hindrance and acetylation effects control the number of available free amine groups and their accessibility to reversibly swell chitin. In addition to volume changes due to acetylation or deacetylation, the presence of free amine groups accelerates the ability of chitin to confer rapid volume change on hydration or on dehydration with water. This is a permeability change to water that reflects a transition from hydrophobic to hydrophilic character. Under constant water content, however, acetylated chitin becomes more crystalline than deacetylated chitin [60].

Depolymerization of chitin is accelerated in amorphous regions because disordered molecules have a more open morphology than the more tightly packed crystalline regions. Stretching the macromolecule by water solvation reduces its modulus [61] and reduces the energy needed to cleave the outer layers of the biopolymer as clarified by Schweiger [62]. Schweiger peeling [63] is most effective when the intercalated acid proton concentration is low enough to avoid long-range steric repulsion effects [64]. Exfoliation occurs as crystalline regions become randomly oriented and physically separated by more than 100 nanometers. Schweiger-peeling effects can extend to become an exfoliation effect when accelerated by mechanical action, chemical depolymerization, and molecular displacement by water-swelling and dissolution effects. Ozone accelerates the Schweiger peeling process by attacking the increasingly exposed O-glycosidic linkages to reduce macromolecular weight. Low molecular weight regions resulting from depolymerization have reduced crystallinity. Some chitin fragments may eventually become sufficiently soluble to be removed by water dissolution as a critical population of deacetylated amine groups becomes labile. The term “denatured chitin” is used here to define short-term morphological change leading to long-term structural and charge-transfer property changes in chitin that act to disrupt its crystalline structure and diminish biological activity in insects.

When the Schweiger-peeling process reaches internal soft tissues, the peeling process is no longer rate-limited by a lack of moisture. Moreover, small voids coalescence and combine into larger voids created through mechanical deformation of gas entrapments, regardless of where they may nucleate. Gas entrapments migrate toward the upper internal surface regions of the insect carapace because gas bubbles are lighter than body fluids. This action forces living biological tissues to decouple and create an abscess within the upper regions of the carapace. Carapace cavities reported in Pacific shrimp, created through exposure to dissolved ozone, are consistent with Schweiger-peeling and chitin exfoliation in the context of denatured chitin.

### The choice of Silver as a Contrasting Agent

Silver is of interest as a forensic contrasting agent for denatured chitin. Voids in chitin are known to incorporate silver [65]. Nitrate anions from silver nitrate form onium-nitrated chitosan plus chelated silver counter-ions at neutral pH. Silver is a high atomic mass transition element that bonds with ammonium ions in favor of alkali earth metals such as sodium [66]. Silver also has the capability to image oxidative stress by forming complex counter-ions with partial negative-charged oxidation products. Silver cations can form complex counter-ions with foreign anions such as [ONON]^-^ if these foreign anions are present in chitin.

Selectivity is desirable in obtaining good contrast due to subtle material property differences. Healthy chitin ordinarily contains high crystallinity. High crystallinity chitin does not make charge-transfer complexes with transition metal salts [67]. Silver (or platinum, which is not used here due to high cost), is not adsorbed by healthy, crystalline, hydrophobic chitin except at pH<2, which indicates high selectivity compared with other transition metals. Other transition metals may bond better with chitin, but with better bonding there is also less selectivity to minor differences in chitin morphology and charge-transfer state.

Low crystallinity or amorphous chitin easily binds to metal ions extracted from water solution [68] and adsorbs or chelates at least 13% silver from dilute aqueous solution near neutral pH [69]. Silver anions form a chemical charge-transfer complex capable of detecting signs of oxidized or denatured chitin. Silver nitrate will also react with amine hydrochloride salts if these are present in chitin, to deposit insoluble silver chloride.

### Case Study Objective

Silver-complex formation with amines and insoluble silver deposition with chloride and other halides can be used to locate and characterize denatured regions in insoluble natural chitin. This assay is unlike methods that rely on slightly soluble and low concentrations of soluble extracted chitin for assay and characterization. The primary aim of this case study is to demonstrate the capability of computer tomographic x-ray radiography to distinguish the location of healthy chitin from denatured or chemically damaged chitin without destroying or dissolving these structures in honey bees, by using the high atomic mass and x-ray scattering properties of silver.

## Results

**Figure 1:**
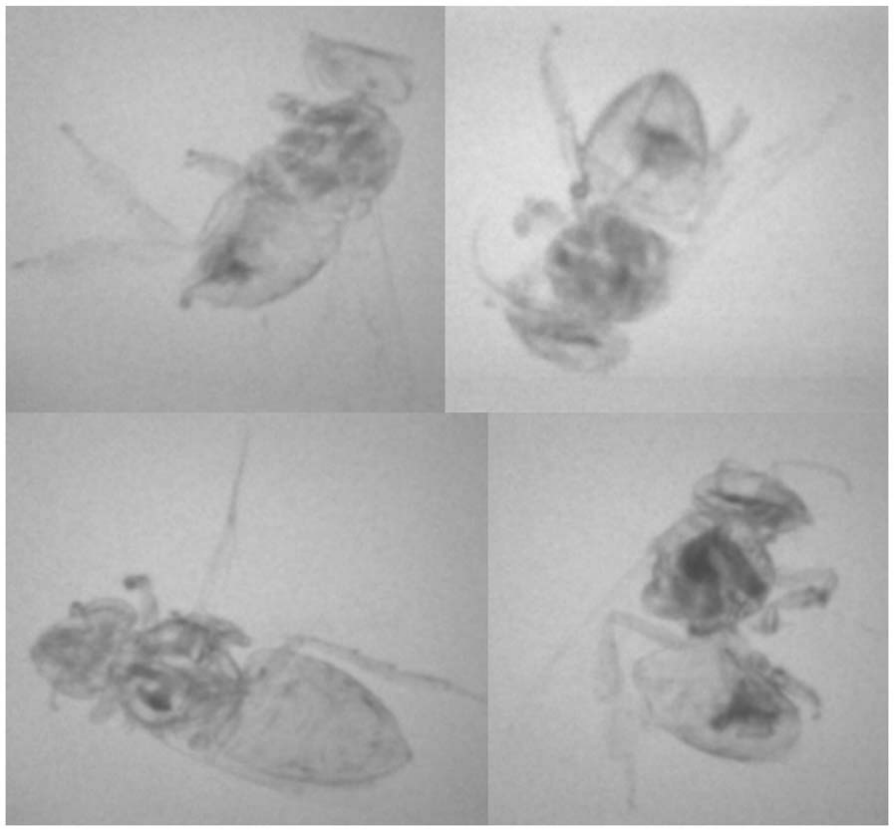
2-Dimensional x-ray micrograph of worker bees used in method development.

**Figure 1** shows conventional two-dimensional x-ray results with darkened areas representing concentrated regions of silver by x-ray absorption. Silver nitrate reaction with hydrophilic, swelled, and deacetylated regions in chitin provided indications of denatured chitin in wing attachments, and upper leg areas at the thorax of different bees. In the lower right bee, there is a darkened band at the dorsal rim of the compound eye. These preliminary results provided sufficient evidence to justify performing micro-CT.

**Table 1** identifies the specimen identification number and treatment of bees, which appear together using similar orthogonal views in the subsequent figures. Each bee was imaged by micro-CT in constant contrast. Greater silver concentration corresponds with greater reflective white regions from scattered x-rays. Supplementary volumetric X-axis and Y-axis rotation movies are provided for each bee identified by specimen number. Each movie was sequentially concatenated from individual micro-CT volumetric scans to convey three-dimensional structural detail.

**Table 1:** Honey Bee Identification.

Bee	Treatment prior to silver nitrate imaging
1	Removed cuticle coating with no environmental exposure (Hatchling bee, baseline reference)
2	Removed cuticle coating and etched with dilute HCl
3	Removed cuticle coating
4	Maintained cuticle coating as a control

**Figure 2:**
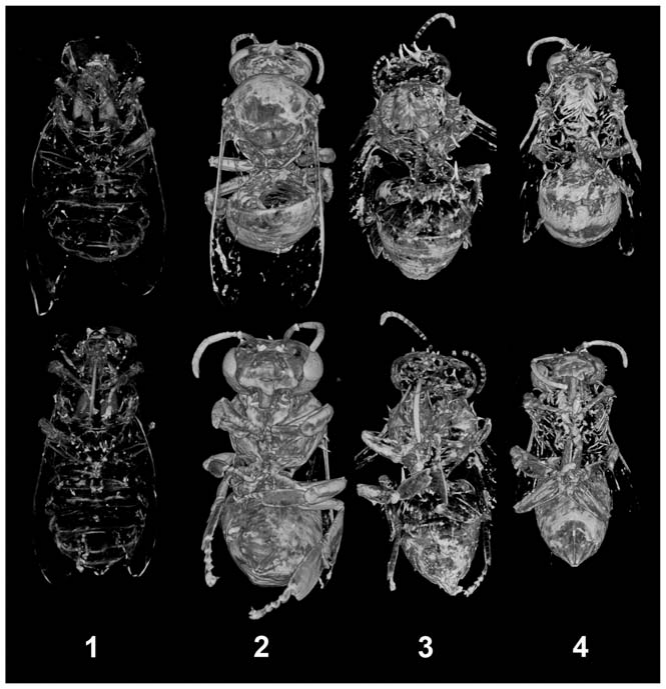
Micro-CT volumetric integration: dorsal and ventral views.

**Figure 2** shows the dorsal view of each bee in the upper row. Newly emerged bee 1 has the least reactivity with silver nitrate, consistent with the hypothesis that Schweiger-peeling has not taken place in this insect because it was not exposed to the environment. The faint whitening of a region in one compound eye observed in bee 1 was tentatively assigned to abrasion damage during emergence, but this origin was not explored for confirmation. Angled views of the slightly white region observed in the volumetric X-axis rotation in **Movie S1** indicate this region is at the surface of the complex eye. The tissues of the internal peritrophic membranes, and the air sacs in the tracheal region were imaged for bee 1 in the y-axis rotation **Movie S2**. Worker-bee 2 has the greatest contrast with silver nitrate, forming silver chloride, or silver as a counter-ion with chitin amine-hydrochloride formed in the acid treatment. It is notable that the compound eye surfaces have reacted to be covered uniformly with bound silver. The chloride containing acid-etch result is representative of silver reacted with glucosamine hydrochloride. The center right portion of the abdomen of worker-bee 2 contains an incompletely peeled layer of chitin that reflects highly and may be a result of acid treatment damage because part of this material, seen in **Movie S3**, is still attached to the external tissues of that bee. Worker-bee 2 provides high contrast imaging of most all chitin features, but the acid treatment has etched away all of the important hairs used to collect pollen; see **Movie S4**. The air sacks do not appear consistently imaged in the three dimensional views of some of the sampled honey bees. Worker-bees 3 and 4 do not show air sacs, yet worker-bees 1 and 2 show them clearly. The preponderance of silver in the head and abdomen of control bee 4 indicates fatty substances not removed by the alcohol treatment has reacted with silver; this difference is attributed to silver complex formation with mono-ester and carboxylic acid in the insect cuticle coating. These sites are diminished or absent in the head and the anterior abdomen of worker-bee 3 treated with the alcohol wash intended to solvate and remove cuticle coating. Notably, the insoluble hairs on the thorax, head, and upper legs of worker-bee 3 are observed to retain a strong affinity for silver.

The lower row of ventral views in **Figure 2** show the greatest silver concentrations were imaged at the joints, at the leading edge of wings, and at the antennae of worker-bees 2, 3, and 4. Environmentally exposed worker-bee 3 shows the greatest concentration of silver in the thorax and the end tip of the abdomen. The compound eyes of bee 3 are mostly transparent to x-rays. This indicates that these eyes were probably well protected by the cuticle coating of this bee, compared with other body regions. The control bee 4 supports this interpretation of the result, as the mono-esters coating the compound eyes demonstrate a notable silver uptake even though the concentration over the eyes appears mottled in the ventral view. This mottling in bee 4 is unlike the uniform reaction observed in the compound eye of the acid-etched bee 2, and is interpreted to represent regions of unequal concentration of the mono-esters covering the compound eyes. The edges of chitin plates of newly emerged bee 1 are barely visible. The diffuse nature of deposited silver in bee 1 conveys a ghostly, glass-like image with few sites of concentrated silver when compared with the rest of the worker-bees. The ventral view shows overlapping chitin folds and leg joints in worker-bees 2, 3, and 4 that may be related to chitin rubbing and wear.

**Figure 3:**
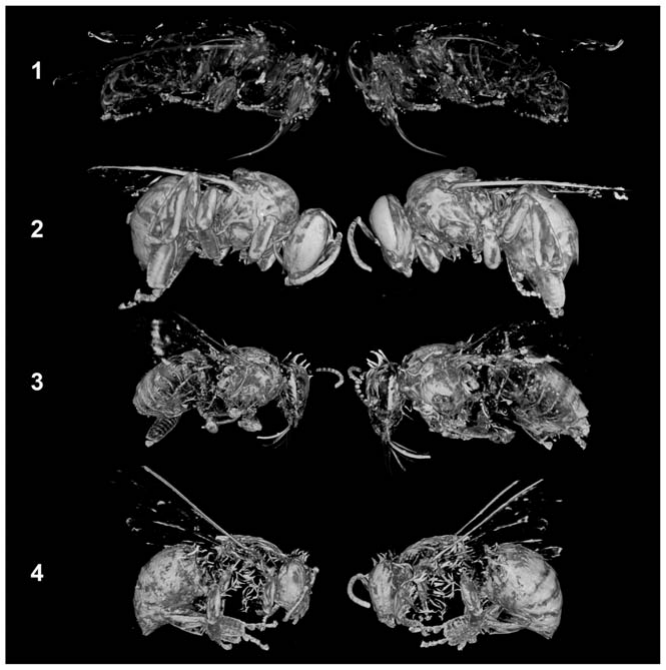
Micro-CT volumetric integration: left and right profile views.

**Figure 3** shows no hairs were imaged by silver nitrate in the upper row volumetric profile views of newly emerged bee 1. Unlike worker-bee 2 where the hairs have been dissolved by dilute acid, visible inspection clarified that hairs do exist on bee 1 but these are not imaged in the x-ray of the newly emerged bee. This result indicates that some environmentally induced morphological or chemical change is responsible for the high chemical affinity for silver-complexes in foraging honey-bee hairs. The profile views of bee 3 indicate that the chitin present in the upper body region was generally more able to complex with silver than the chitin in the lower regions. Because cuticle coating was removed from bee 3, some susceptibility to silver reaction may be tentatively associated with chitin exposed to the sun or the environment arising from above the insect. This transition can also be seen by comparing the upper and lower regions of bee 3 in **Movie S5** and **Movie S6**.

Control bee 4 shows silver heavily reacts with compounds present in the waxy mono-esters and carboxylic acids of the cuticle coating, as seen in **Movie S7** and **Movie S8**. This heavy silver-complex deposition result was used to assign a probable mixed signal contribution of molecular species complex with silver in cuticle coating plus molecularly charged chitin to the total X-ray response. This result supports the imaging of insects or regions of insects having intact-cuticle to compare with those insects or regions of insects having solvent removed cuticle coating prior to imaging. Both types of preparation appear to provide complementary and therefore useful information.

**Figure 4:**
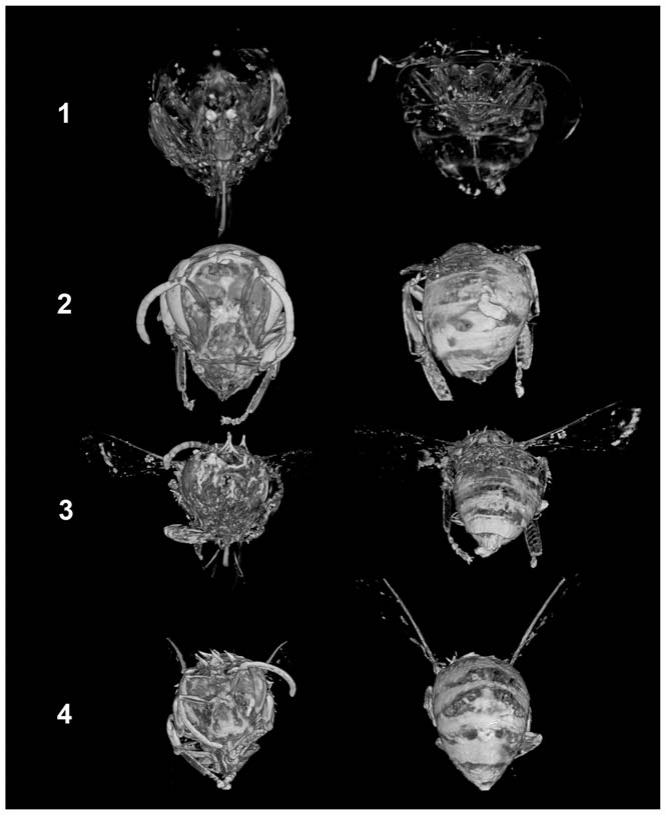
Micro-CT volumetric integration: anterior and posterior views.

The base of the antennal attachment to the head in **Figure 4** is a region of high silver-complex forming ability in each bee imaged by micro-CT. This result appears even in the newly emerged bee 1 that had no exposure to the environment outside the hive. The conserved result of high silver deposition in a specific local tissue in each case studied indicates some consistent morphology, molecular charge composition, or mechanical wear had a reproducible and significant contribution to the observed silver imaged in chitin. The conserved or least-variable result in these tissues may indicate a possibly useful reference location for the assignment of an internal standard or internal reference value to rate, ratio, or otherwise evaluate those tissues in other imaged regions of the insect that are apparently influenced more by environmental variability.

**Figure 5:**
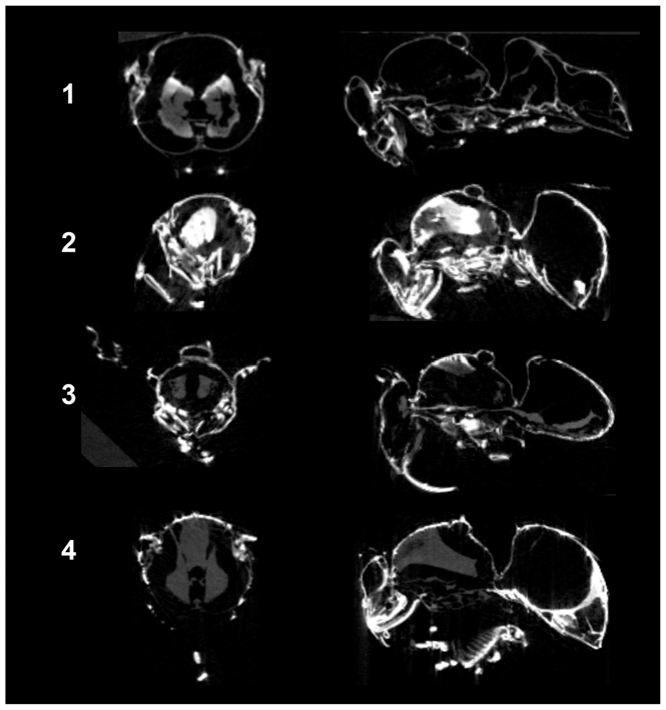
Micro-CT transverse section at wings, longitudinal section at mid-plane.

**Figure 5** provides a computer tomographic transverse section view taken at the point of the wing attachment in each bee, and a longitudinal section view taken along the mid-plane of each bee. The strongest internal silver signals arise in acid treated worker-bee 2, but with different structures than bee 4 with cuticle coating of mono-esters not removed by solvent treatment. The high silver concentration within the internal thorax of worker-bee 2 suggests internal acid damage is linked to easily nitrated internal tissues. Undigested fats or fatty linings in the gut may cling to the inner wall of the peritrophic membranes; this may explain the appearance of stained tissues loose in the abdominal cavities. Some of these may also be tracheal system parts. It is noted that tissue displacement by natural drying may introduce an artifact that can complicate a conclusive assignment in these section views.

## Discussion

Chitin regions with hydrophilic character increased by the Schweiger-peeling mechanism will preferentially attract, swell, and react with aqueous silver nitrate. Reaction of sterically non-hindered amine in chitin using silver nitrate leaves silver-complexes by the mechanism of counter-ions formed with silver. The creation of silver chloride after dilute hydrochloric acid etch of chitin is chemically similar to a two stage environmental exposure of chitin subjected to low pH non-chloride acid rain followed by a reaction with chloride present in ground dust or sea salt fogs to form the chitin amine hydrochloride. Subsequent exposure of chitin to silver nitrate forms the insoluble silver chloride precipitate at the location of the chloride ion. This creates a chemical basis for the biochemical determination of in-situ chitin imaging in the macromolecular structure of killed and prepared insects.

The base of the antennal attachment is a flexible chitinous joint, and is imaged as a site of concentrated silver in all bees of this case study. This organ may require chitin of low crystallinity and a high degree of deacetylation to function properly. Bee 1 with no environmental exposure expressed similar concentrated silver deposits at the same location on the head, as other bees. This result implies the antennal base is a natural artifact of enhanced silver migration into soft chitin tissues in honey bees, and may not be a peculiar site of chemically induced damage.

Compared with aged bees, newly emerged bees are more deformable; they have a low modulus and low crystallinity chitin that may ease solution diffusion and penetration of silver ions into the tracheal system and air sacs. Flexible tissues may deform more readily than inflexible tissues on swirling different bees with different properties in aqueous solution. This may vary the introduction of silver ions into the tracheal system by fluid displacement. It was observed that each insect appeared to become softer and more uniformly deformable when in the alcohol solution as compared with the water solution containing silver. Future evaluation of alcohol solvated silver is suggested to uniformly soften tissues and improve the ability of silver to more consistently permeate and image the tracheal system, air sacs, and peritrophic membranes of insects.

Insoluble hairs on the thorax, head, and upper legs of worker-bees are observed to retain a strong affinity for silver. As mentioned in past studies by previous researchers using fluorescent dyes, insoluble fluorescent particles had been found. These particles could not be identified beyond the observation that they were insoluble oxidation products unable to be solvated for assay. When considered within the context of the present results, the strong affinity by silver for honey bee hairs supports a view that silver forms a complex with oxidized hair. If sometimes such hairs may break off to form insoluble particles, this suggests previously unidentifiable fluorescent particles classified as insoluble oxidation products may, in fact, consist of honey bee hair fragments.

### Potential Implications

The global nature of honey bee and pollinator declines makes atmospheric variables worthy of broad examination. Air quality and acid rain may be synergistic variables in the limiting dosage consideration of pesticides, microbes, and pest infestation, but have not yet been sufficiently studied in this context. For example, long-term ground level ozone in combination with dilute acidic moisture may be synergistic if the presence of both can oxidize protective mono-esters and carboxylic acids in cuticle coatings.

Waxy or oily cuticle coating substances are permeated into chitin with the highest concentration of waxes and oils on the surface, while some low molecular weight components occupy the interior molecular volume between polymeric oligosaccharide branches. Speculatively, the imaging results may be interpreted as regions where cuticle coating concentrations were reduced prior to denaturing. The protective cuticle coating may not so much expire as become displaced or reduced in concentration at local regions of individual insects. Healthy chitin must remain mostly hydrophobic to maintain good coverage and water repellency by a protective cuticle coating. The formation of excessive amine hydrochloride salts or amine hydrophilic groups may cause a tendency to repel waxy or oily cuticle coating at sites of increasingly hydrophilic chitin.

While silver nitrate reacting with amine hydrochloride was demonstrated for the eye surfaces of the acid chloride treated honey bee, the representative chemistry used to demonstrate the imaging technique may not capture natural variations in deposition that could be influenced by other variables such as local abrasion of chitin, surface roughness due to abrasion, or a static cling effect that favors points of attachment by salt containing aerosols or particles. Environmental exposure may have influenced the image of the compound eye in the lower right bee in **Figure 1**. The darkening of the dorsal rim region of the eye of that insect may represent an artifact of two-dimensional x-ray imaging, or it may be typical of natural defects with micro-inclusions attracting natural salts to bind with silver, or it might be a much more serious preliminary indication of molecular charges with localized surface damage to those corneal lenses of honey bee compound eyes or ommatidium which are specialized for polarized light detection and navigation. Schweiger-peeling effects located at the surface of the compound eye cornea may be related to external chemical attack from acid rain or fog following a reaction with chlorides to form surface hydrochlorides. The silver imaging technique may provide a way to observe serious side effects of ozone or acid rain pollution. The potential implications to agriculture do warrant extensive further study that must go far beyond this first case study to verify both the extent and magnitude of this possibility.

Geographic regions with a high nitric oxide concentration and regions with a high ozone concentration are often widely separated in the troposphere by either physical location or time of day. Either of these ROS gases may have quite different chemical effects on chitin, especially when combined with exposure to ultraviolet light. Ozone depolymerizes chitin and reduces its molecular weight. Trace nitric oxides may form a gaseous intercalant with chitin that accelerates loss of crystalline order by promoting aqueous intercalation on exchange with water by hydrophilic swelling. Speculatively, these two effects may not always be independent if the combination of different exposures of nitric oxide and ozone obtained by movement of bees from one locality to another may act together to increase the rate of Schweiger-peeling effects in the chitin of peritrophic membranes. Chitin subjected to volume changes may have reduced crystallinity due to wetting and drying. Increased polar properties and higher permeability of chitin to water may reduce resistance to both gram positive and gram negative microbes, which rely on a polar surface to perform attachment. The implication is that silver imaging may help identify changes in the properties of the peritrophic membrane as a general indicator of chemical and structural compromise internal to the insect that may impact the ability to digest food safely. This technique may be useful to complement existing research to identify a particular biological or biochemical origin of peritrophic breakdown.

More development of the present work is required in a scaled-up statistical study with narrowly focused environmental exposures capable of obtaining a case-by-case proof-of-concept refinement of this technique before it can become a prescriptive method for chitin denaturing.

Care must be taken not to use the results of the present exploratory case study to interpret a causal link between a particular insect disorder cause and effect. A range of ideas can be suggested for casual or longitudinal studies using silver staining with X-ray CT imaging as part of a larger experimental framework. For example, exposure to genetically modified pollen of crops containing enhanced levels of natural or introduced insecticides may alter or damage the internal chitin microfibrils critical to digestion in the peritrophic membranes of some kinds of beneficial insects such as honey bees as well as that of targeted agricultural pests.

In another potential application of the technique, it may be of interest to search for an abscess in the honey bee that correlates with a similar kind of abscess reported in pacific shrimp according to ozone dosage exposure in “soft-shell syndrome.” Modification of the present method to use glycol such as ethylene glycol or another polar organic solvent with low vapor pressure should improve resistance to drying and shrinkage of internal tissues in this type of work while maintaining solubility with silver nitrate contrasting agent.

Many pathological combinations could relate to a reduction of cuticle coating and subsequent chitin chemistry or property change. The imaging method shown by the results of the present exploratory case study provides support for the further development of this technique in future honey bee and insect pollinator research. In colony collapse, young brood larvae and aged insects remain alive as worker bee populations decline. Protective mono-ester cuticle coating production on chitin peaks in worker bees at 15 days, whereas larvae and aged insects tend to produce long chain hydrocarbon coatings instead. Speculatively, chemical or environmental attack may alter the distribution, quality, or hydrophobic coverage of the cuticle coating on the most exposed foraging insects. Alteration of the availability of long chain waxy materials in stressed foliage may also contribute to a fundamental dietary deficiency related to a reduction of cuticle coating efficacy in pollinating insects. Some or of all these effects may then lead to denaturing of chitin, which can now be observed in specific regions of the insect. Reduction of cuticle coating molecular length quality or quantity may affect the denaturing of chitin, leaving stressed insects prone to become more susceptible to disease. Future work may clarify if this appears first in middle-aged foraging bees, as compared with brood or aged bees. Longitudinal studies using micro-CT imaging techniques, pathogen classification, and waxy raw material availability are suggested to assist in obtaining a better global understanding of pollinating insect stress as well as colony collapse disorder in farmed honey bees.

Other analysis should be used to complement the technique of the present case study in future studies. The biological molecular stability of insect chitin preserved in alcohol or other media for long periods of time will require clarification to help develop a mature science of insect molecular imaging. Infra-red analysis is a non-destructive qualitative assay capable of creating a qualitative map of external chitin with deacetylated chemical properties in insects. A possible correlation may exist between infra-red surface maps of deacetylation versus a more three-dimensional map of denatured chitin imaged by x-ray micro-CT. Statistical evaluations of individual bees using these or similar techniques may help to identify precursor conditions leading to pathogen susceptibility in insects.

As introduced, there are many reactions capable of denaturing chitin, but gaseous pollutants and acidic rains are of unusually special interest due to geographically high concentrations produced within the industrialized nations. An in-situ three-dimensional chemical marker method using silver nitrate was shown to be capable of imaging the cuticle coating and denatured chitin. Chitin imaging by silver, and cuticle coating imaging before and after an alcohol solvent extraction, are expected to help identify and rank the importance of potential environmental sources of chitin denaturing by providing signals that can be easily seen in individual bees by x-ray imaging.

### Conclusions

A denatured chitin and compromised cuticle coating hypothesis of compromised insect immune defense due to environmental exposure, was proposed. Denatured chitin is associated with deacetylation, loss of crystallinity, hydrophilic swelling, oxidation, Schweiger-peeling, and hydrochloride salt formation. Reactivity to silver nitrate in honey bees by micro-CT imaging provided encouraging results. Silver was concentrated in flexible joints of all bees and particularly in the peritrophic envelope and membranes, glossa, and hairs of foraging honey bees. Apparent chemical damage to insect vision by acidic attack of surface chitin in compound eyes was inferred by greatly enhanced silver deposits on the eye cornea after a brief and mild acidic exposure. Silver appeared to be diffuse in a newly emerged honey bee not exposed to environments outside the hive cell. Differences in x-ray images of bees after alcohol immersion were associated with the removal of cuticle coating by solvent prior to staining and development with silver.

Agricultural extensionists and researchers interested in biomacromolecule-ligands may consider this technique for future evaluation in creating a database of “snapshots” of denatured chitin and cuticle coating health in the structures of individual honey bees exposed to environmental chemicals to supplement existing research with a visual record of these effects. Cuticle coatings and molecularly charged chitin regions were demonstrated capable of accepting a silver stain. Cuticle coating was removed using alcohol solvent, leaving regions of molecularly charged chitin capable of accepting a silver stain. These or similar imaging techniques can help to validate the appropriate targeting of new pesticides, to help determine the health of cuticle coatings in stressed insects, or to help categorize typical imaging characteristics for standardized or limiting dose exposures. The imaging methods described in this report may also find use to support existing agricultural research using images of bees before and after pathological conditions arise in controlled longitudinal studies associated with evidenced based honey bee CCD, or in a comparative epidemiology of insect pollinators in areas of greater and of lesser population decline.

## Materials and Methods

Silver Nitrate, 99.95% purity was obtained from Salt Lake Metals, Utah, USA. Through-transmission images were obtained using a model FXE-160 x-ray microscope, FineFocus Röntgen-Systeme GmBH, at 60 KV, and 2 micro amps.

Micro-CT scan was performed on a Siemens model Inveon CT, at 80 KV, at 180 micro-amps, 2000 microseconds per degree per slice, 33 minutes total scan time, using Admira 5.3.1 software with DICOM reader 5.3 and Skeleton Option 5.3, from Visage Software.

In the method development, silver nitrate concentration and soak time were adjusted to obtain reasonable contrast using a two-dimensional x-ray. Non-neutral pH may deacetylate chitin; therefore the use of a more typical pH 9.5 ammoniacal silver nitrate was avoided.

The newly emerged bee 1 was cut out of a hive cell to represent the case of chitin that was never exposed to environmental influences outside of the comb. The rest of the bees are adult honey bee workers of unknown age. Each bee specimen except a control bee number 4 was treated with 91% isopropanol for 30 minutes to remove the cuticle coating containing mono-esters and carboxylic acids capable of reacting with silver nitrate. Alcohol saturated bees were dried with gentle tumbling for solvent removal. A control (worker-bee 4) was not treated with isopropanol to control the effect of silver-complex reaction with the natural coating intact. Next, worker-bee 2 was treated with 1% hydrochloric acid etch for 90 seconds, followed by several rinses with distilled water. This case was representative of some water-soluble pesticides that may have chemically active chloride as part of their reactive composition. Bee 3 was a worker-bee treated the same as newly emerged bee 1. Each bee was then immersed into aqueous 30% silver nitrate dissolved in pH neutral distilled water for 12 hours in darkness at 22 degrees C. Each bee was then removed from the nitrate solution, rinsed in distilled water, and blotted dry. Silver was developed in the bees by gentle tumbling under a 470 nm dental curing lamp for 10 minutes.

## Supporting Information

Table S1 ****
(DOC)

Movie S1 **Bee 1, X-axis rotation.**
(MPG)

Movie S2 **Bee 1, Y-axis rotation.**
(MPG)

Movie S3 **Bee 2, X-axis rotation.**
(MPG)

Movie S4 **Bee 2, Y-axis rotation.**
(MPG)

Movie S5 **Bee 3, X-axis rotation.**
(MPG)

Movie S6 **Bee 3, Y-axis rotation.**
(MPG)

Movie S7 **Bee 4, X-axis rotation.**
(MPG)

Movie S8 **Bee 4, Y-axis rotation.**
(MPG)
