# Supplementary material for: Micro-CT Imaging of Denatured Chitin by Silver to Explore Honey Bee and Insect Pathologies
Source: PLoS One. 2011 Nov 16;6(11):e27448. doi: 10.1371/journal.pone.0027448 (PMC3217983; doi:10.1371/journal.pone.0027448)
Supplement: Table S1 — (DOC) [file pone.0027448.s001.doc]

## Table 1 Honey Bee Identification

| Bee | Treatment prior to silver nitrate imaging |
| --- | --- |
| 1 | Removed cuticle coating with no environmental exposure  (Hatchling bee, baseline reference) |
| 2 | Removed cuticle coating and etched with dilute HCl |
| 3 | Removed cuticle coating |
| 4 | Maintained cuticle coating as a control |
